# In Vitro Antiproliferative Effect of Cannabis Extract PHEC-66 on Melanoma Cell Lines

**DOI:** 10.3390/cells12202450

**Published:** 2023-10-13

**Authors:** Ava Bachari, Nazim Nassar, Srinivasareddy Telukutla, Roby Zomer, Chaitali Dekiwadia, Terrence J. Piva, Nitin Mantri

**Affiliations:** 1 The Pangenomics Lab, School of Science, RMIT University, Bundoora, VIC 3083, Australia; ava.bachari@rmit.edu.au (A.B.); srinivasareddy.telukutla@rmit.edu.au (S.T.); 2 School of Health and Biomedical Sciences, RMIT University, Bundoora, VIC 3083, Australia; naz.nassar@rmit.edu.au (N.N.); terry.piva@rmit.edu.au (T.J.P.); 3 Faculty of Health, Charles Darwin University, Casuarina, NT 0810, Australia; 4 MGC Pharmaceuticals Limited, West Perth, WA 6005, Australia; roby@mgcpharma.co.uk; 5 RMIT Microscopy and Microanalysis Facility, STEM College, RMIT University, Melbourne, VIC 3000, Australia; chaitali.dekiwadia@rmit.edu.au; 6 UWA Institute of Agriculture, The University of Western Australia, Perth, WA 6009, Australia

**Keywords:** melanoma, skin cancer, cannabinoids, *C. sativa*, PHEC-66

## Abstract

Melanoma, an aggressive form of skin cancer, can be fatal if not diagnosed and treated early. Melanoma is widely recognized to resist advanced cancer treatments, including immune checkpoint inhibitors, kinase inhibitors, and chemotherapy. Numerous studies have shown that various *Cannabis sativa* extracts exhibit potential anticancer effects against different types of tumours both in vitro and in vivo. This study is the first to report that PHEC-66, a *Cannabis sativa* extract, displays antiproliferative effects against MM418-C1, MM329 and MM96L melanoma cells. Although these findings suggest that PHEC-66 has promising potential as a pharmacotherapeutic agent for melanoma treatment, further research is necessary to evaluate its safety, efficacy, and clinical applications.

## 1. Introduction

Melanoma, characterized by its high metastatic rate and resistance to traditional treatments, is the leading cause of death in skin cancer cases [1]. While melanoma represents only 5.5% of skin cancer diagnoses, it accounts for >80% of related fatalities [2]. Melanoma arises from melanocytes in the epidermis and typically appears in irregularly shaped moles [3]. Factors contributing to its development include chronic inflammatory conditions such as psoriasis and eczema [4], genetic predisposition [5], and prolonged exposure to environmental hazards [6], particularly UV radiation, which can directly damage melanocyte DNA [7]. Additionally, melanoma as a malignant neoplasm can result from the activation of oncogenes combined with the suppression/inactivation of tumour suppressor genes, typically originating in epithelial melanocytes [8,9].

Swiftly spreading and invading surrounding tissues, progressing to lymph nodes and metastasizing to other organs [10,11,12], malignant melanoma (MM) requires early diagnosis and prompt treatment for optimal patient outcomes [13]. In recent years, various pharmacotherapeutic approaches have been utilized to treat melanoma. The mitogen-activated protein (MAP) kinase transduction pathways approach inhibits the MAPK signalling pathway using agents such as dabrafenib, vemurafenib, binimetinib, encorafernib, trametinib, selumetinib, and cobimetinib; this is one of the mainstream therapies that is utilized in melanoma treatment [14,15,16,17,18,19,20,21,22,23,24]. These agents bind to BRAF and MEK, which are vital components of the MAPK signalling pathway, making them critical treatment strategies for patients with BRAF-mutant melanoma [25]. Additionally, traditional immune therapies such as tumour necrosis factor-alpha (TNFα) [26], interleukin-2 (IL-2) [27], interferon-gamma (IFNγ) [28], and immune checkpoint inhibitors (ICIs) such as pembrolizumab, nivolumab, atezolizumab, ipilimumab, and relatlimab are central to melanoma treatment, and are also utilized intensively in this field [29,30,31,32,33].

Thus far, immune checkpoint inhibitors, including the anti-programmed cell death antigen-1 (anti-PD1) and anti-cytotoxic T lymphocyte antigen-4 (anti-CTLA-4), have shown promise in treating melanoma [34]. Nevertheless, several drawbacks, including significant adverse drug effects, have been engendered by such treatments, hindering patient compliance. Moreover, these immunotherapeutic agents can also lead to a cancer relapse and post-treatment drug resistance, further limiting their efficacy [35,36]. Furthermore, the options for treating metastatic melanoma remain limited, with patients experiencing a median survival time of only 6–9 months and a 3-year overall survival rate of <15% [37].

Researchers and clinicians have collaborated over the last few years to explore the potential of natural cannabis extracts and synthetic equivalents as unconventional remedies for treating melanoma. Cannabinoids, including endocannabinoids, phytocannabinoids, and synthetic cannabinoids, are known to signal the cell via CB1 and CB2 receptors, which are part of the G protein-coupled receptor (GPCR) system [38]. They can influence the immunogenic tumour microenvironment by initiating antitumorigenic cell signalling, controlling apoptosis and angiogenesis, and modulating the release of various immunogenic and inflammatory cytokines [39,40]. For instance, Cannabidiol (CBD), a non-psychotic cannabis extract, has shown promise in blocking the cell cycle of gastric cancer cells and inducing apoptosis in breast cancer cells through the endoplasmic reticulum stress pathway [41,42]. Building upon those achievements, further endeavours have been undertaken to enhance treatment alternatives. Consequently, this research assesses the impact of the *Cannabis sativa* extract PHEC-66 on different melanoma cell lines (MM418-C1, MM329 and MM96L) regarding its antiproliferative effects. The findings from this study may provide a basis for future investigations into the anti-melanoma capabilities of PHEC-66, potentially establishing it as a promising phytocannabinoid extract with anticancer properties.

## 2. Materials and Methods

### 2.1. Materials

RPMI-1640 media, heat-inactivated foetal bovine serum (FBS), streptomycin, and penicillin, were obtained from Thermo Fisher Scientific (Melbourne, Australia). 3-(4,5-Di methylthiazol-2-yl) 2,5-diphenyltetrazolium bromide (MTT), annexin V-FITC (Thermofisher, 11-8005-74) and PI suspended in annexin-binding buffer (Thermofisher, 00-6990-42) were purchased from Thermofisher (Melbourne, Australia). The propidium iodide flow cytometry kit (Abcam139418) was purchased from Abcam (Melbourne, Australia), while the cellTiter-Glo^®^ Luminescent was purchased from Promega (Melbourne, Australia).

Human melanoma MM418-C1 (primary (1°) melanoma possessing the oncogenic BRAF^V600E^ mutation), MM329 (1° melanoma possessing wild type BRAF (BRAF^WT^)) and MM96L (metastatic or secondary (2°) melanoma possessing the oncogenic BRAF^V600E^ mutation) cells, human epithermal melanocytes (HEM), human immortalized keratinocytes (HaCaT), and neonatal human dermal fibroblasts (NHDF) were used in this study. MM418-C1, MM329, MM96L, and HaCaT cells were kindly supplied by Prof Nichola Hayward and Peter Parsons, Queensland Institute of Medical Research, Brisbane, Australia. NHDF cells were purchased from Promocell (Banksia Scientific, Brisbane, Australia).

### 2.2. Methods

Cells were cultured in RPMI-1640 tissue culture medium supplemented with 10% (*v*/*v*) FBS and 1% (*v*/*v*) penicillin and streptomycin. The cells were incubated at 37 °C in a humidified 5% CO_2_ incubator and passaged every 3–4 days until they reached 80–90% confluency.

#### 2.2.1. MTT Assay

The cytotoxicity of the test compounds in MM418-C1, MM329, and MM96L cells was evaluated using the MTT assay. These cells were seeded into 96-well plates at a density of 3000–10,000 cells per well, depending on their doubling times, and were allowed to adhere for 24 h at 37 °C and 5% CO_2_. After 24 h incubation, the spent tissue culture medium was replaced with a fresh tissue culture medium containing the test compounds dissolved in DMSO. DMSO (≤0.06% *v*/*v*) was also tested as the solvent control. After 48 h incubation, the medium containing the test compounds was aspirated, 100 µL of culture media containing 5 mg/mL MTT was added to each well, and the cells were further incubated for 3 h in the dark at 37 °C. At the end of this period, the media containing MTT was removed, and 100 µL DMSO was added to each well to solubilize the crystallized formazan product. The plates were read on a microplate reader at 570 nm wavelength. The % growth inhibition was calculated as 100 − [(Mean O.D. of the treated cell × 100)/Mean O.D. of vehicle-treated cells (DMSO)]. The IC_50_ values were calculated using GraphPad Prism 8 software. All measurements were performed in triplicate.

#### 2.2.2. Colony Formation Inhibition Assay

MM418-C1, MM329, and MM96L cells in the exponential growth phase were plated into 6-well culture plates at a single cell density (300 cells/well) and allowed to adhere for 24 h. After that, the cells were incubated with fresh tissue culture medium containing a PHEC-66 at a concentration equal to 50%, 100%, and 200% of their respective IC_50_ values. After 24 h, this medium was replaced with a fresh tissue culture medium, and the cells were incubated for seven days, and the medium was replaced every two days. After that, the cells were washed with 1 mL PBS (pH 7.4), fixed with 4% paraformaldehyde, and stained with 0.5% (*w*/*v*) crystal violet (in 80 mL distilled H_2_O and 20 mL methanol) for 30 min. The cells were then rinsed with distilled water to remove excess dye. Plates were photographed with a Nikon Coolpix 950 digital camera.

#### 2.2.3. In Vitro Cell Migration Assays

MM418-C1, MM329, and MM96L cells (5 × 10^5^ cells/well) were cultured in 12-well plates for 24 h to form a confluent monolayer. After that, a sterile 200 µL pipette tip was used to scratch the monolayers. In order to remove non-adherent cells, the wounded monolayers were washed twice with 1 mL PBS (pH 7.4). Next 1 mL fresh tissue culture media containing PHEC-66 at a concentration equal to 50%, 100%, and 200% of their respective IC_50_ values was added to the cells. Using a phase contrast microscope, cells that had migrated across the wound were imaged at the same three or selected fields at 0, 24, and 48 h post-treatment.

#### 2.2.4. Ultra-Structural Analysis of Melanoma Cells Using Transmission Electron Microscopy (T.E.M.)

Melanoma cells were treated with PHEC-66 at a concentration equal to 50%, 100%, and 200% of their respective IC_50_ values for 48 h. The treated cells were then washed with 1 mL PBS and primary fixed with 2.5% (*v*/*v*) glutaraldehyde and 2% (*v*/*v*) paraformaldehyde in 0.1 M cacodylate buffer (pH  7.3) for 1 h. The fixed cells were centrifuged (400×  *g* for 5 min at RT), rinsed with 0.1 M sodium cacodylate buffer (pH  7.3) twice, and left overnight in the same buffer. The cells were post-fixed with 1% (*w*/*v*) osmium tetroxide and 1.5% (*w*/*v*) potassium ferrocyanide for 1.5 h at RT and then washed twice with distilled water for 10 min. Dehydration was conducted as follows; 50% (*v*/*v*) ethanol for 15 min, followed by 70% (*v*/*v*) ethanol for 15 min, 90% (*v*/*v*) ethanol for 15 min, 95% (*v*/*v*) ethanol for 15 min, 100% (*v*/*v*) ethanol twice for 30 min before being washed with 100% (*v*/*v*) acetone for 30 min. Infiltration was carried out using a mixture of acetone and Spurr’s resin mix (1:1) on a shaker overnight at RT. The next day, fresh acetone: Spurr’s resin mix (1:1) was added to the cells and left for 2 h. Then, 100% Spurr’s resin was added to the cells before they were placed under a vacuum for 2 hours. Finally, the cells were cured at 70 °C for 48 h. Ultrathin sectioning was carried out with LeicaUltracut UCTultra-microtome (Leica Biosystems, Mount Waverley, Australia) to produce 90 nm–100 nm thin sections. The TEM grids containing sections were examined under a JEOL1010 transmission electron microscope equipped with a Gatan Orius SC600 CCD Camera (Gatan, Pleasanton, CA, USA).

#### 2.2.5. Determination of the Effect of PHEC-66 Treatments in 3D Multicellular Spheroids

This three-dimensional (3D) multicellular spheroid (MCS) model mimics the in vivo pathophysiology of tumour tissue by replicating the phenotypic diversity, nutrient, and oxygen gradients, and micro-metastases with gene expression that occurs during the clinical manifestation process [43,44].

The 3D multicellular spheroids were established by centrifuging these cell lines in ultra-low attachment (ULA) plates for one day before treating them with different concentrations of PHEC-66. The morphological changes in the MCSs were observed after 48 h of treatment with PHEC-66. Here, we utilised the CellTiter-Glo 3D Cell Viability Assay, a validated method for assessing cell viability in 3D microtissue cultures. After a 48 h incubation period, we added 100 μL of CellTiter-Glo^®^ 3D Reagent to the medium containing cells. The contents were vigorously mixed for 5 min to induce cell lysis. Proper mixing is crucial for the efficient extraction of ATP from 3D microtissues. The plate was allowed to incubate at room temperature for an additional 25 min to stabilise the luminescent signal. Finally, the luminescence was measured using a plate reader.

Adding PHEC-66 to the cells at a concentration greater than their IC_50_ value resulted in a significant reduction in the surface area and volume of the spheroids compared to the corresponding control. These results were similar to those observed in the 2D cell monolayer assays.

### 2.3. Statistical Analysis

The results were analysed via one-way analysis of variance (ANOVA) using GraphPad Prism software (version 8). For normally distributed data, the means were compared using the one-way analysis of variance (ANOVA) and Tukey’s post hoc test. Statistical values of *p* < 0.05 were considered significantly different. The inhibitory concentration (IC_50_) of PHEC-66 for cytotoxicity was derived from a nonlinear regression model (curve-fit) based on a sigmoidal dose–response curve (variable) and computed using GraphPad Prism version 8.

## 3. Results

### 3.1. Cell Viability Assay

**Figure 1:**
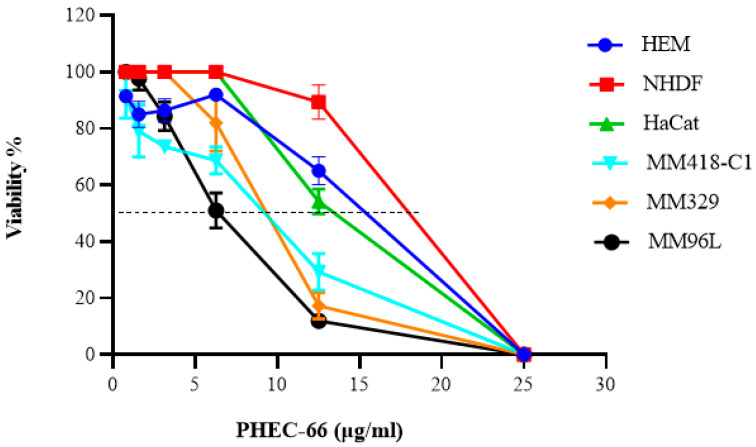
Effect of PHEC-66 on the viability of cultured skin cells. PHEC-66 ranged between (1.56 and 25 µg/mL) and was added to the cells for 48 h. Data represent the mean values ± standard deviation of three independent experiments performed in triplicate. GraphPad Prism was used to produce the dose–response curve and IC_50_ doses.

PHEC-66 reduced the viability of the human melanoma cell lines (MM418-C1, MM329 and MM96L) in a concentration-dependent manner (Figure 1). Moreover, it also decreased, to a lesser extent, the viability of HEM, NHDF, and HaCaT cells as well. However, this effect was only observed at concentrations greater than 13 µg/mL, which is almost twice the IC_50_ concentration observed for the melanoma cells. The IC_50_ values for PHEC-66 on the various cell lines are shown in Table 1.

As seen in Table 1, the melanoma cells were significantly more sensitive to PHEC-66 compared to non-transformed skin cells.

### 3.2. Colony Formation Assay

**Figure 2:**
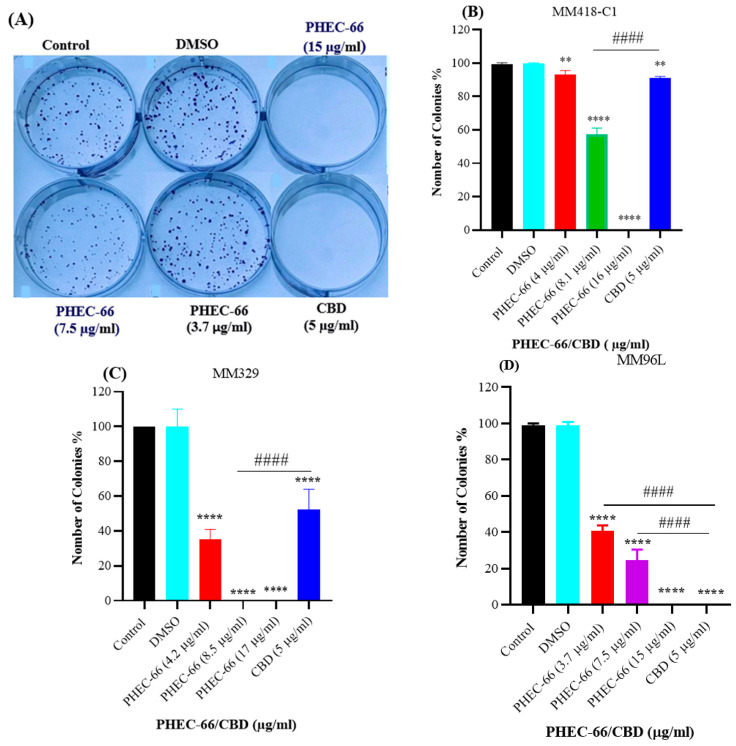
PHEC-66 and CBD reduces the formation of melanoma cell colonies. A representative image of MM96L cell colonies formed post-PHEC-66 and CBD treatment (**A**). The effect of PHEC-66 and CBD on the colonies formed by (**B**) MM418-C1, (**C**) MM329, and (**D**) MM96L cells following exposure to PHEC-66 and CBD. PHEC-66 concentrations added to each cell line were 50%, 100%, and 200% of the respective IC_50_ values, while CBD was equivalent to that present within PHEC-66 at its IC_50_ value. Asterisks represent statistically significant differences between PHEC-66 or CBD-treated cells compared to the control group (** *p* = 0.005, **** *p* ≤ 0.0001). Hash represents statistically significant differences between CBD-treated cells compared to PHEC-66 treated cells (#### *p* ≤ 0.0001). All data represent the mean ± SEM of three independent experiments.

In order to study the impact of PHEC-66 on colony formation, we conducted experiments on various melanoma cell cultures, such as MM418-C1, MM329 and MM96L cells. As PHEC-66 contains 60% cannabidiol (CBD), the effect of this compound on its own at the corresponding concentration found in the extract was also tested on these cell lines. The concentrations of PHEC-66 used in the experiment were 50%, 100%, and 200% of their respective IC_50_ values for that cell line, as shown in Table 1. After melanoma cells were treated with different concentrations of PHEC-66 and CBD, they were sorted and re-seeded to form colonies over 7 days of incubation, as seen in Figure 2A. The treatment administered to all cells reduced colony formation in a dose-dependent manner (Figure 2B–D). Nevertheless, when treated with twice the IC_50_ concentration of PHEC-66, colony formation was eliminated in all cell lines tested (Figure 2B–D).

Interestingly, when CBD is used at the same concentration as the IC_50_ of PHEC-66, it had a lower inhibitory effect on colony formation in both MM418-C1 and MM329 cells (Table 2). However, in the case of MM96L cells, both PHEC-66 at its IC_50_ concentration and of CBD caused 100% cell death (Table 2).

### 3.3. In Vitro Cell Migration Assays

**Figure 3:**
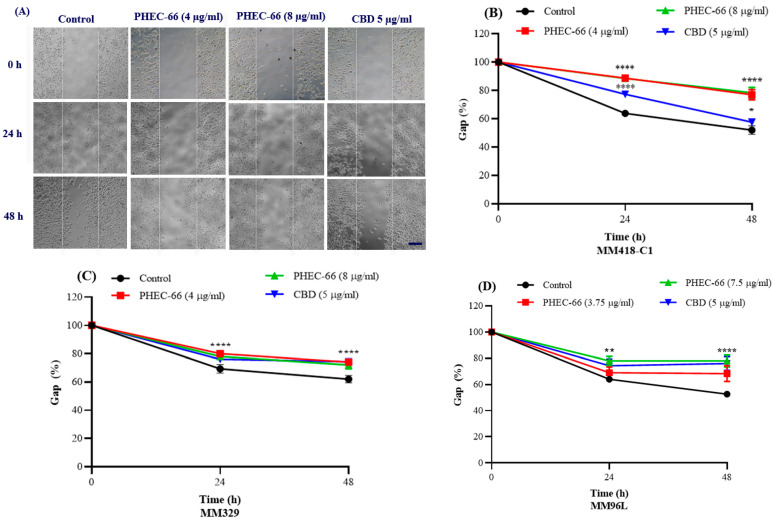
PHEC-66 and CBD reduces the motility of melanoma cells. A representative image of MM418-C1 gap closure over 48 h following PHEC-66 and CBD treatment (**A**). The effect of PHEC-66 (50% and 100% of their respective IC_50_ values) and CBD (equivalent to the amount present in 100% PHEC-66) treatment for 48 h on the migration of MM418-C1 (**B**), MM329 (**C**), and (**D**) MM96L cell lines (**D**). Error bars indicate ± SEM (*n* = 3). Asterisks indicate statistically significant differences between CBD-treated cells and PHEC-66 treated cells (* *p* = 0.05, ** *p* = 0.01, **** *p* < 0.0001 one-way ANOVA with Tukey’s multiple comparisons test). (Scale bar = 100 µm).

A scratch assay was performed to examine the ability of PHEC-66 to attenuate melanoma cell motility. In this experiment, the melanoma cell lines were incubated in the presence of PHEC-66 and CBD for 48 h at three different time points (0, 24, and 48 h). As noted above, PHEC-66, at twice its IC_50_ concentration, caused complete cell killing and was excluded from this study. Therefore, PHEC-66 at only 50% or 100% of its IC_50_ concentration was added after the monolayer was scratched. Analysis of the impact of PHEC-66 on cell migration was performed on captured images of the cultured cells under light microscopy (Figure 3A).

The gap closure for each cell line was measured within the marked boundaries, as shown in Figure 3A. Compared to the control groups, we observed a significant decrease in the gap closure in the presence of PHEC-66 and CBD as early as 24 h post-treatment.

Following the administration of PHEC-66 and CBD, all tested cell lines, including MM418-C1, MM329, and MM96L, demonstrated a noticeable decrease in the rate of wound healing compared to the control groups. This decrease was evident in the reduced closure of the wound gap between the edges of the targeted cell lines. After 24 h, PHEC-66 reduced the closure of the wound gap by 27% in the MM418-C1 cells when compared to the untreated controls (Figure 3B). However, its effect on the gap closure for the treated MM329 and MM96L cells was less than that seen for the MM418-C1 cells (8% and 15%, respectively), when compared to the untreated controls. Interestingly, CBD had a reduced effect on retarding the gap closure in only the MM418-C1 cells (16%) when compared to the untreated controls. The inhibitory effect of CBD on gap closure in the MM329 and MM96L cells (8% and 11%, respectively), was similar to that exerted by PHEC-66 (Figure 3C,D).

### 3.4. Morphological Changes

**Figure 4:**
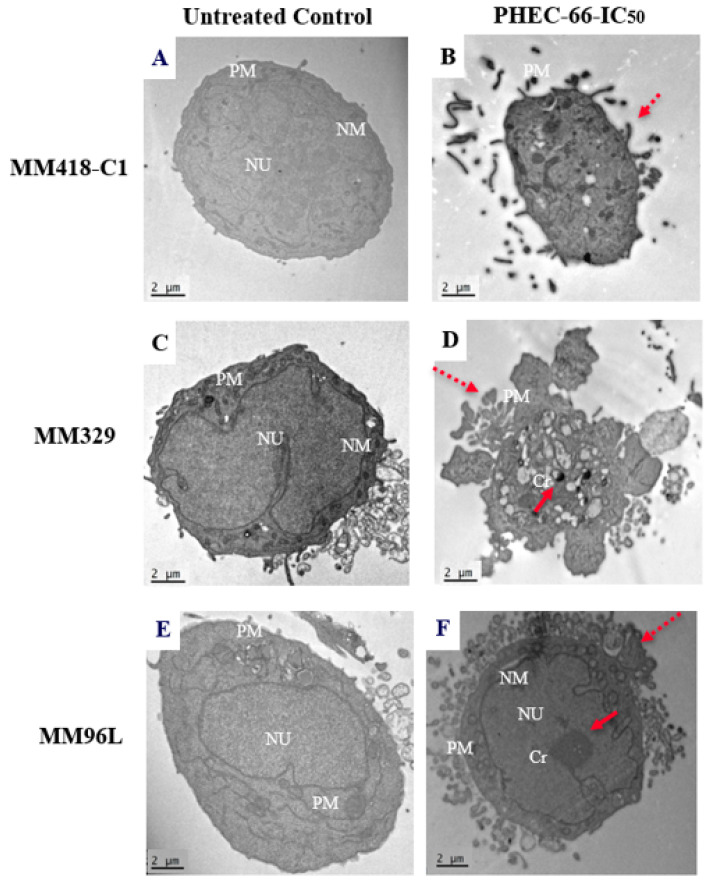
Effect of PHEC-66 on the morphology of MM418-C1, MM329, and MM96L melanoma cells. The cells were treated with the PHEC-66 at their respective IC_50_ concentrations for 48 h and then examined under TEM. Solid-line arrow: chromatin condensation, dotted-line arrow: cell membrane blebbing, NU: nucleus, NM: nuclear membrane, Cr: chromatin condensation, PM: plasma membrane. (**A**) MM329 control cells, (**B**) PHEC-66-treated MM329 cells, (**C**) MM418-C1 control cells, (**D**) PHEC-66-treated MM418-C1 cells, (**E**) MM96L control cells, (**F**) PHEC-66-treated MM96L cells (Scale bar = 2 µm).

We observed the effect of PHEC on the morphology of the melanoma cells as observed under transmission electron microscopy (TEM). PHEC-66 at its IC_50_ concentration caused changes to the morphology of these cell lines (Figure 4). These cells showed distinct changes compared to the untreated control group that exhibited normal morphology with an intact nucleus and plasma membrane (Figure 4A,C,E).

Following PHEC-66 treatment, significant morphological changes were observed, as evidenced by the chromatin condensation (Figure 4B), protrusion of the plasma membrane or blebbing, and cell shrinking (Figure 4D,F), which are indicative of both necrosis and cell apoptosis [45]. In contrast, the untreated cells exhibit typical characteristics with a well-defined nucleus, prominent nucleoli, and an absence of condensed DNA at the periphery (Figure 4A,C,E).

### 3.5. Morphology and Growth of 3D Spheroids Following Treatment with PHEC-66

Multicellular 3D spheroids are highly representative of the in vivo pathophysiology observed in tumour tissues, particularly in terms of the heterogeneity of phenotypes, nutrient and oxygen gradients, and micro-metastases [46]. These spheroids exhibit gene expression profiles that more closely resemble the clinical expression profiles of tissue. Compared to cells grown in 2D monolayers, tumour cells growing in 3D spheroids experience different adhesive, mechanical, and topographical forces [47]. Moreover, by establishing tumour cell 3D spheroids, researchers can replicate the cell–cell and cell-ECM interactions that occur in solid tumours. In a larger context, in vitro 3D tumour cell cultures are a more appropriate preclinical model for screening cancer drug leads for in vivo solid tumours.

**Figure 5:**
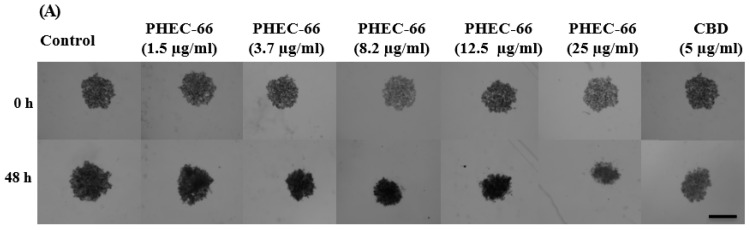

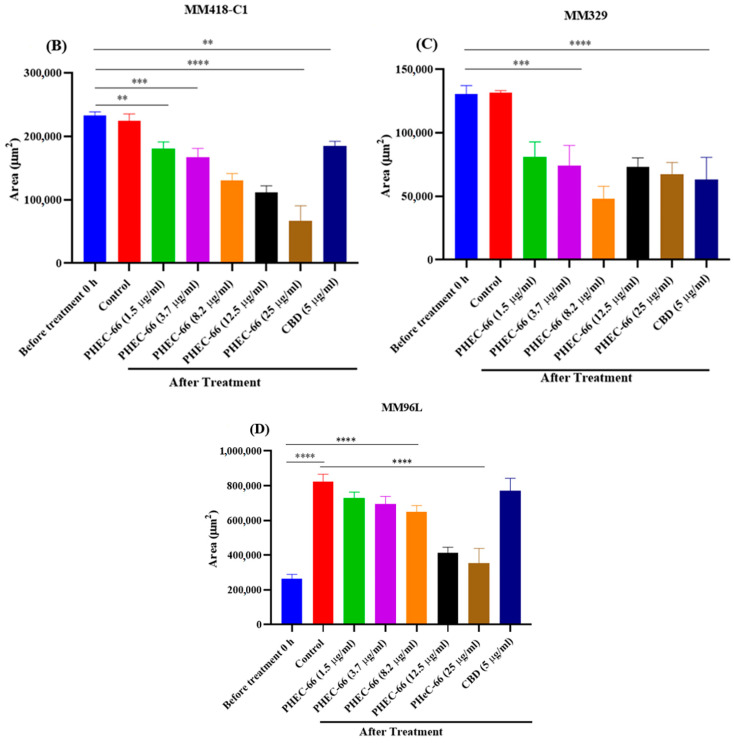
PHEC-66 and CBD treatment reduces the surface area of melanoma cell spheroids. A representative image of the effect of 48 h PHEC-66 and CBD treatment on MM96L spheroids (**A**). The top panel is of the spheroids at 0 h, and the bottom panel is after 48 h treatment. The spheroids were exposed to PHEC-66 and CBD for 48 h and changes in their surface area can be seen in MM418-C1 (**B**), MM329 (**C**), and MM96L cells (**D**). Asterisks indicate statistically significant differences between untreated spheroids and PHEC-66-treated spheroids (** *p* = 0.01, *** *p* = 0.001, **** *p* < 0.0001 one-way ANOVA with Tukey’s multiple comparisons test). The results expressed are the mean ± SEM (*n* = 3) (Scale bar = 900 μm).

To confirm the cytotoxic effects of PHEC-66 in the in vivo tumour environment, 3D multicellular spheroids were created by cultivating MM418-C1, MM329, and MM96L cells in wells of an ultra-low attachment (ULA) plate for 24 h. Approximately 5000 cells from the above cell lines were placed in ULA 96-well plates to form spheroids with a diameter of ~900 μm. The growth of these spheroids was observed during the initial stage. Afterward, the treated spheroids were subjected to varying concentrations of PHEC-66. Microscopic observations of the spheroids are shown in Figure 5A. The effect of PHEC-66 and CBD on the surface area of the MM418-C1, MM329, and MM96L spheroids are calculated using Image J version 1.53 t (Figure 5B–D).

PHEC-66 caused a significant dose-dependent reduction in the spheroid surface area, which was similar to the effect it had on cell proliferation (Figure 5A–C), consistent with the results observed in the 2D cell monolayer assays. Furthermore, when CBD was administered at an equivalent concentration to that found in the IC_50_ of PHEC-66, a notable decrease in the morphological size of all the treated cell lines was observed. However, this reduction was less than that elicited by PHEC-66 itself.

### 3.6. Viability of Melanoma Cell Lines in 2D and 3D Cell Cultures

**Figure 6:**
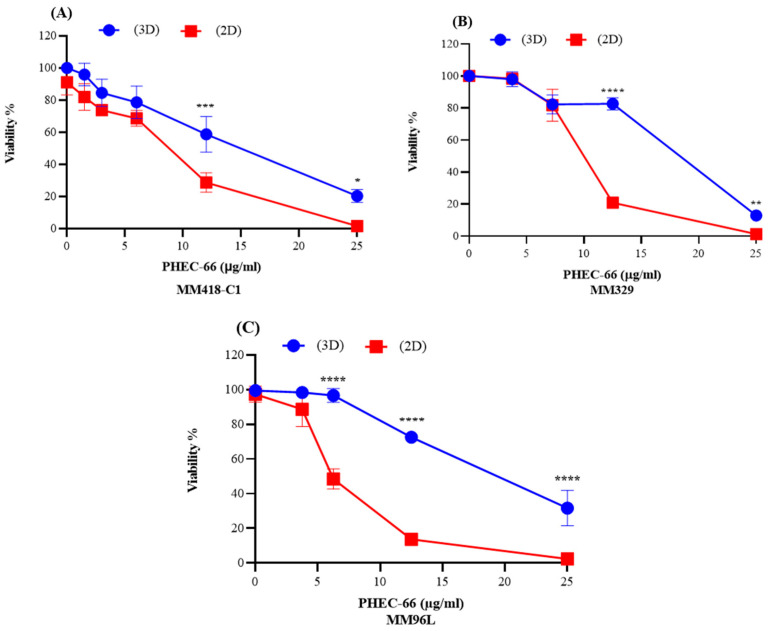
PHEC-66 elicits a greater cytotoxicity to cells grown in 2D monolayers vs. 3D spheroids. PHEC-66 was added to 2D and 3D cultures of MM418-C1 (**A**,**B**) MM329 (**B**), and MM96L cells (**C**) for 48 h. Error bars indicate ± SEM (*n* = 3). Asterisks indicate statistically significant differences between 2D- and 3D-treated cells with PHEC-66 treated cells (* *p* < 0.05, ** *p* < 0.005, *** *p* = 0.001, **** *p* < 0.0001 one-way ANOVA with Tukey’s multiple comparisons test).

To obtain a more precise representation of the physiological conditions that exist within a living organism, we compared the viability of 3D melanoma cell cultures (MM418-C1, MM329, and MM96L) with those grown in 2D cell monolayers. Here, PHEC-66 at its IC_50_ concentration was added to both types of cultures, and their viability was recorded at 48 h, as seen in Figure 6.

The IC_50_ value for PHEC-66 was significantly higher (1.82–2.6-fold) in the 3D spheroids than in the 2D cultures, as seen in Table 3.

## 4. Discussion

Melanomas generally have significant tumour heterogeneity and one of the highest mutation frequencies. For example, in BRAF^V600E^, one of the most frequently observed mutations in melanoma, glutamic acid replaces valine at codon 600, resulting in a mutated protein that promotes uncontrolled cell growth [48]. This mutation is observed in 40–50% of melanoma cases [48].

Targeted therapies, such as BRAF inhibitors, have been developed to impede the activity of BRAF^V600E^ and related mutations to improve patient outcomes. However, resistance to such pharmacotherapies can develop over time [49,50]. Therefore, ongoing research aims to find additional treatment strategies and combinations to overcome resistance and enhance the effectiveness of treatments for melanoma patients with these mutations. In light of this, we undertook a study investigating the potential efficacy of the *Cannabis sativa* extract, PHEC-66, on a range of melanoma cell lines. The study has revealed that PHEC-66 reduced the viability of MM418-C1 (BRAF^V600E^) and MM96L (BRAF^V600E^) and MM329 (1° BRAF^WT^) cells, in a dose-dependent manner. Both MM418-C1 and MM329 cells were derived from primary melanomas found in a non-chronic sun-damaged lesion [51], while MM96L cells were taken from a secondary melanoma found in the lymph node [52]. As PHEC-66 consists of ~60% CBD, the effect of this cannabinoid was tested on its own at the concentration that was present in the respective IC_50_ values for PHEC-66.

In vitro assays were conducted to assess the therapeutic potential of PHEC-66 on these melanoma cell lines. The IC_50_ value of PHEC-66 for melanoma cells was shown to be approximately half of that observed in non-transformed cells, particularly that of HEM. The melanoma cells exhibited increased sensitivity to PHEC-66, leading to decreased cell viability, indicating that this extract may potentially hinder the growth of melanoma cells at concentrations that do not significantly impact a number of different non-transformed skin cells (HEM, HaCaT, and NHDF cells).

A colony formation assay was used to evaluate the rate at which the cells formed colonies in response to PHEC-66 treatment. At its IC_50_ concentration, PHEC-66 extract exerted a considerable inhibitory effect on colony formation in MM418-C1, MM329 and MM96L cell lines after a seven-day treatment period, ranging from 43% in MM418-C1 to 100% in MM329 and MM96L cells. These findings suggest that PHEC-66 can effectively suppress the ability of these cells to form colonies, indicating its potential as a therapeutic agent for inhibiting melanoma cell growth and proliferation.

The scratch assay evaluates the rate of cell proliferation and migration by observing the ability of the cells to close a scratch on a cell monolayer. A wound-healing assay was utilized to quantify the migratory cells within predetermined boundaries for each cell line. There was an approximate 20% reduction in gap closure following PHEC-66 treatment compared to the untreated controls. These observations were consistent across the three cell lines within 24 h following the induced surface scratch wound. This effect could be derived by activating the Rho family proteins resulting in the reduction of RhoA GTPase activity, followed by a loss of actin/myosin microfilaments and a consequent reduction in cell migration capacity [53,54]. However, further investigations, such as rescue experiments involving the introduction of exogenous RhoA GTPase or its activators into cells [54], are required to verify if Rho family proteins are involved in this process.

The complexities of cell migration and metastasis involve multiple factors, including changes in the expression and activity of cell adhesion molecules like integrins, cadherins, and selectins [55,56]. These molecules mediate interactions between cells and the extracellular matrix or neighbouring cells, influencing cell migration [56]. Studies have indicated that cannabinoids can modulate the expression and function of these adhesion molecules [57,58]. For instance, CBD has been observed to upregulate epithelial markers such as E-cadherin and downregulate mesenchymal markers such as N-cadherin in cancer cells, reducing cell migration and invasion [59]. Cell migration is also influenced by various signalling pathways, such as the inhibition of cyclic adenosine monophosphate (cAMP) or the activation of protein kinase B (Akt) signalling [60,61]. Through the manipulation of downstream pathways, cannabinoids have been observed to modulate the expression and activity of effectors such as growth factors, including VEGF (vascular endothelial growth factor) and EGF (epidermal growth factor) [62,63].

Furthermore, the physical properties of the extracellular matrix, such as its stiffness and composition, can also have an impact on cell migration [64]. Cells exhibit different migratory abilities depending on the stiffness and composition of the matrix they interact with. While the direct effects of cannabinoids on the physical properties of the extracellular matrix are not well documented, they can indirectly influence the matrix through various mechanisms. For example, cannabinoids can regulate the expression and activity of matrix metalloproteinases (MMPs) involved in ECM remodelling [65]. By modulating MMPs, cannabinoids may indirectly affect the composition and structure of the ECM, thereby influencing cell migration [65,66]. Additionally, cannabinoids possess anti-inflammatory and immunomodulatory effects, indirectly impacting the ECM [67]. Inflammation plays a role in ECM remodelling, and by modulating inflammation via elevation of proinflammatory cytokines, e.g., L6 and tumour necrosis factor-alpha (TNFα) [68], cannabinoids may indirectly affect the physical properties of the ECM by reducing these pro-inflammatory effectors and subsequently influence cell migration [67].

To summarize, the effect on cellular processes, MMPs, soluble factors, and inflammation collectively contribute to potential indirect changes in the ECM, potentially influencing cell migration. Therefore, further research is necessary to comprehend these relationships and understand the specific mechanisms involved.

The multicellular spheroids assay is an additional in vitro model utilized to investigate the effect of PHEC-66 on melanoma cells. Microscopic observations revealed distinct morphological characteristics of the 3D spheroids formed by the MM418-C1, MM329 and MM96L melanoma cells. PHEC-66 was shown to reduce (a) the size (surface area) of the spheroid significantly and (b) increase the number of dead cells in all cell lines, consistent with the findings from the 2D cell monolayer experiments. The cytotoxic effect of PHEC-66 on the 3D melanoma spheroids was less than that seen on the 2D cultures. The IC_50_ effect of PHEC-66 on the 3D cultures was approximately twice that seen for the 2D cultures of the melanoma cell lines.

The area of the cell surface exposed to PHEC-66 is significantly higher in 2D monolayers than in 3D spheroids. This disparity in exposure levels may contribute to reduced cell viability in the 2D environment, characterized by more constrained conditions for cell growth [69]. In addition to that, it is essential to note that cells in 3D models may respond differently to PHEC-66 treatment than in 2D models due to their interactions with the extracellular matrix and neighbouring cells [70]. In the spheroids, drug access can be limited by a more extensive extracellular matrix surrounding the cells, making it more difficult for such agents to penetrate the cells than those growing in 2D monolayers [71].

In light of those mentioned above, drug resistance in 2D culture can be attributed to the dynamic cellular interactions between neighbouring cells governed by the extracellular matrix that influences cellular decision-making processes [72]. In 3D culture systems, cells form spherical aggregates or spheroids within a matrix, suspension medium, or on a matrix, unlike in 2D monolayer cultures. This type of culture (3D spheroids) promotes natural cell-to-cell and cell-to-ECM interactions, resulting in a cell morphology that closely resembles that seen in vivo [73]. Moreover, the cells in 3D spheroids can exist at different development and metabolic stages, including proliferative, quiescent, apoptotic, hypoxic, and necrotic cells, creating cellular heterogeneity that mimics tissues found in vivo, including tumours [74]. The outer layers of spheroids are primarily made up of viable, proliferating cells, while these cells in the core receive fewer nutrients, oxygen, and growth factors from the medium, making them quiescent or hypoxic, leading to death [72]. Consequently, the cellular processes of cells grown in 3D culture closely resemble those seen in vivo due to the similarity in cell morphology and interactions.

It is worth noting that this study found that the metastatic melanoma cell line, MM96L, was more responsive to PHEC-66 treatment than the primary tumour cell lines, MM418-C1 and MM329. This difference could be attributed to several possible factors, including (1) the possibility that metastatic cancer cells expression of higher levels of these receptors in secondary cancer cells compared to primary cancer cells, making them more sensitive to the effects of cannabinoids [75], (2) differential regulation of signalling pathways. In other words, the activation of cannabinoid receptors can modulate various intracellular signalling pathways, leading to inhibition in cell proliferation and initiating apoptosis [76]. Metastatic cancer cells, such as MM96L, may have distinct patterns of signalling pathway activation compared to primary cancer cells, leading to a differential response to cannabinoid treatment. (3) heterogeneity of cancer cells, i.e., cancer cells can display a high degree of heterogeneity, with different subpopulations exhibiting varying degrees of treatment resistance [77]. The results suggest the possibility that metastatic cancer cells used in the experiment may have exhibited increased responsiveness to PHEC-66 extract, potentially due to the existence of a subgroup that was particularly sensitive to this form of treatment. (4) Differences in experimental conditions are another factor that might be behind these differences. It is important to note that differences in the experimental conditions could influence the observed differences in responses between metastatic and primary cancer cells, such as cell culture media, drug concentrations, or treatment duration. Overall, the reasons for the observed differences in treatment response would need to be investigated more to fully understand why the metastatic cancer cells were more responsive to PHEC-66 than primary cancer cells.

Considering the above, the PHEC-66 has demonstrated a noticeable cytotoxic effect on various melanoma cells. The transmission electron microscopy (TEM) analysis revealed distinct cellular changes, including membrane protrusion or blebbing, and cell shrinkage, characteristic of apoptosis [45]. Inhibiting MAPK signalling pathways and modulating metabolic processes, such as the significant accumulation of reactive oxygen species, are crucial cellular targets commonly employed by novel pharmacotherapeutics. Specifically, cannabinoids have been shown to induce apoptosis through these mechanisms. Therefore, further investigations are warranted to elucidate the underlying mechanism of action through which PHEC-66 acts as an antiproliferative agent and how it activates signalling pathways that in turn can trigger cell death in melanoma cells.

## 5. Conclusions

In conclusion, the results of this study demonstrate that PHEC-66 extract derived from *Cannabis sativa* exerts a significant cytotoxic effect on MM418-C1, MM329, and MM96L melanoma cell lines while having a lesser effect on human keratinocytes (HaCaT), human epidermal melanocytes (HEM), and normal human dermal fibroblasts (NHDF). Although the mechanism of PHEC-66’s anti-melanoma activity remains unknown, this study suggests it may induce apoptotic and necrotic cell death pathways. Further research is necessary to fully comprehend the underlying mechanisms of PHEC-66’s actions and assess its potential as a natural source of anticancer compounds.

## Figures and Tables

**Table 1:** Comparison of the IC_50_ value for cells treated with PHEC-66. Data represent the mean values ± standard deviation of three independent experiments performed in triplicate.

Cell Lines	IC_50_ (μg/mL)
NHDF	17.23 ± 0.98
HaCaT	13.37 ± 1.90
HEM	15.71 ± 1.32
MM418-C1	8.21 ± 0.75
MM329	8.47 ± 0.14
MM96L	7.41 ± 0.94

**Table 2:** Effect of PHEC-66 compared to CBD on colony formation of different melanoma cell lines. PHEC-66 was applied at its IC_50_ concentration, while CBD was added at its equivalent concentration to that present in PHEC-66.

Cells	Colony Inhibition at IC_50_ of PHEC-66	Colony Inhibition at an Equivalent Concentration of Pure CBD
MM418-C1	43%	9%
MM329	100%	48%
MM96L	100%	100%

**Table 3:** Comparison of the IC_50_ values of PHEC-66 for melanoma cells grown in either 2D or 3D cultures.

Cell Type	PHEC-66 IC_50_ (2D Cultures)	PHEC-66 IC_50_ (3D Cultures)	Ratio (3D/2D)
MM418-C1	8.21 µg/mL	15.00 µg/mL	1.82
MM329	8.73 µg/mL	19.44 µg/mL	2.23
MM96L	7.40 µg/mL	19.28 µg/mL	2.61

## Data Availability

Not applicable.
